# Cocrystal structure of meningococcal factor H binding protein variant 3 reveals a new crossprotective epitope recognized by human mAb 1E6

**DOI:** 10.1096/fj.201900374R

**Published:** 2019-10-05

**Authors:** Federica Bianchi, Daniele Veggi, Laura Santini, Francesca Buricchi, Erika Bartolini, Paola Lo Surdo, Manuele Martinelli, Oretta Finco, Vega Masignani, Matthew J. Bottomley, Domenico Maione, Roberta Cozzi

**Affiliations:** * GlaxoSmithKline, Siena, Italy;; † University of Florence, Firenze, Italy;; ‡ GlaxoSmithKline, Rockville, Maryland, USA

**Keywords:** *Neisseria meningitidis*, fHbp, human antibodies, vaccine

## Abstract

The 4 component meningococcus B vaccine (4CMenB) vaccine is the first vaccine containing recombinant proteins licensed for the prevention of invasive meningococcal disease caused by meningococcal serogroup B strains. 4CMenB contains 3 main recombinant proteins, including the *Neisseria meningitidis* factor H binding protein (fHbp), a lipoprotein able to bind the human factor H. To date, over 1000 aa sequences of fHbp have been identified, and they can be divided into variant groups 1, 2, and 3, which are usually not crossprotective. Nevertheless, previous characterizations of a small set (*n* = 10) of mAbs generated in humans after 4CMenB immunization revealed 2 human Fabs (huFabs) (1A12, 1G3) with some crossreactivity for variants 1, 2, and 3. This unexpected result prompted us to examine a much larger set of human mAbs (*n* = 110), with the aim of better understanding the extent and nature of crossreactive anti-fHbp antibodies. In this study, we report an analysis of the human antibody response to fHbp, by the characterization of 110 huFabs collected from 3 adult vaccinees during a 6-mo study. Although the 4CMenB vaccine contains fHbp variant 1, 13 huFabs were also found to be crossreactive with variants 2 and 3. The crystal structure of the crossreactive huFab 1E6 in complex with fHbp variant 3 was determined, revealing a novel, highly conserved epitope distinct from the epitopes recognized by 1A12 or 1G3. Further, functional characterization shows that human mAb 1E6 is able to elicit rabbit, but not human, complement-mediated bactericidal activity against meningococci displaying fHbp from any of the 3 different variant groups. This functional and structural information about the human antibody response upon 4CMenB immunization contributes to further unraveling the immunogenic properties of fHbp. Knowledge gained about the epitope profile recognized by the human antibody repertoire could guide future vaccine design.—Bianchi, F., Veggi, D., Santini, L., Buricchi, F., Bartolini, E., Lo Surdo, P., Martinelli, M., Finco, O., Masignani, V., Bottomley, M. J., Maione, D., Cozzi, R. Cocrystal structure of meningococcal factor H binding protein variant 3 reveals a new crossprotective epitope recognized by human mAb 1E6.

*Neisseria meningitidis* is an exclusively human pathogen, able to colonize the mucosal surfaces, proliferate, and (under some instances) invade the bloodstream, causing morbidity and mortality in infants, children, and young adults worldwide (1–3). Bacterial strains have been classified in 12 serogroups based on the composition of their capsular polysaccharide (4, 5), but only serogroups A, B, C, W, X, and Y are responsible for almost all cases of invasive meningococcal disease (1, 2, 6).

To survive in the human host, the meningococcus has evolved several strategies to evade bactericidal killing, such as capsular polysaccharides, which mimic human cell components or by the expression of proteins are able to recruit immune system inhibitors (7). Indeed, whereas several capsular polysaccharide–based vaccines against serogroups A, C, W, and Y have been developed and licensed (8–10), the capsular polysaccharide of meningococcal serogroup B (MenB) is composed of polymers of α (2–8)-linked *N*-acetylneuraminic acid, which is also found in many tissues, especially in the CNS, the human neural cell adhesion molecule, and is therefore not considered a suitable vaccine target due to the risk of autoimmunity (11).

Two vaccines specific for MenB [termed 4 component meningococcus B vaccine (4CMenB) and bivalent rLP2086] have been developed and are licensed for use in numerous countries worldwide. One of the main antigens included in both vaccines is the factor H binding protein (fHbp), a 28-kDa membrane-anchored surface lipoprotein able to bind the human factor H (hfH) protein, one of the most abundant serum negative regulators of human complement. Recruitment of hfH to the meningococcal surface helps the bacteria to evade the complement-mediated killing and renders them more resistant to the immune system (12, 13).

To date, more than 1000 distinct amino acid sequences of fHbp have been identified, and they can be divided into 3 variant groups (v1, v2, and v3). Within the fHbp variant groups, the sequence identity is usually above 87%; instead, between variant groups, the sequence identity can be as low as 62% (14).

Several three-dimensional (3D) structures of fHbp have been solved by NMR (15) and X-ray crystallography (16–18), and despite sharing amino acid identity as low as 62%, the structures of the fHbp variants 1, 2, and 3 are well conserved and consist of 2 domains. The C-terminal domain adopts a canonical 8-stranded β-barrel conformation, whereas the fHbp N-terminal domain shows a more unusual taco-shaped β-barrel fold characterized by higher intrinsic flexibility. Differential scanning calorimetry profiles showed independent unfolding of the 2 barrels (17–19). The C-terminal β-barrel melts at temperatures above 80°C in all 3 variants; in contrast, the N-terminal β-barrel exhibits highly variable melting temperatures at 70°C in v1, 61°C in v3, and at 37°C in v2 (20). This intrinsic thermal instability and a noted susceptibility to protease cleavage (21) could explain why the structural characterization of the N-terminal domain of wild-type v2 was unsuccessful so far, although the structure of a stabilized mutant form was determined (21). The variability in the amino acid composition between the fHbp variants results in relevant chemical and physical differences that regulate the molecular and dynamic properties of fHbp. All fHbp variants bind to hfH with high affinity, with the equilibrium dissociation constant, *K*_d_, in the nanomolar range, but large differences in the stability of the complexes were observed (22). The crystal structures of domains 6 and 7 of hfH with fHbp v1 and fHbp v3 were determined by X-ray crystallography, confirming that hfH binding to fHbp is mediated by the same region of the protein but through a distinct set of v1 or v3 residues (17, 23). Vaccination with fHbp elicits a robust immune response in mice, rabbits, macaques (14, 24, 25), and humans (26, 27), conferring protection by 2 pathways: directly engaging C1q to activate the classic complement-mediated killing, or blocking hfH binding to the bacteria to increase the susceptibility of the bacterium to killing by the alternative complement pathway (28–31). To date, several anti-fHbp monoclonal antibodies were described, but probably due to the high amino acid sequence variability, the majority of them are variant-specific (14, 32–37). However, a few anti-fHbp antibodies that were able to recognize more than 1 variant were reported (18, 38–40).

In a recent study aiming to characterize the human B-cell repertoire in response to vaccination with 4CMenB, single plasma blasts from 3 vaccinated adults were isolated, and the genes encoding Ig variable regions were sequenced. Human mAbs against fHbp, Neisserial Heparin Binding Antigen, and Neisseria adhesin A were identified (41). The resulting panel of 10 anti-fHbp antibody fragments (Fabs) was analyzed by 2 different research groups, and both teams found 2 anti-fHbp antibodies (1A12 and 1G3) to be crossreactive with the 3 main fHbp variants (41, 42). Moreover, the crystal structure of one of these (1A12) in complex with fHbp v1 was determined, revealing a conserved epitope localized exclusively on the C-terminal β-barrel (18, 41).

As an extension of the first investigations by Giuliani *et al.* (41) and Beernink *et al.* (42), a new longitudinal study is in progress analyzing a much larger repertoire of anti-fHbp human mAbs from 3 additional vaccinees. In this study, 110 human Fabs (huFabs) targeting fHbp and isolated at different time points were produced in *Escherichia coli* and analyzed in terms of antigen binding specificities. Although the 4CMenB vaccine contains the variant 1 form of fHbp, many crossreactive antibodies were identified. Here, in order to better understand the human immune response to 4CMenB and the possibility that immunization with fHbp v1 raises crossreactive antibodies, we present a structural and functional characterization of one of these human antibodies called 1E6 in complex with fHbp v3.

## MATERIALS AND METHODS

### Human samples

Human samples were collected from 3 adults immunized with *Bexsero*, also referred to as 4CMenB, in a clinical trial conducted in Krakow, Poland, approved by the Bioethics Committee of the District Medical Doctors Chamber in Krakow and conducted in accordance with the Declaration of Helsinki. The use of samples was performed upon written informed consent obtained from participants before the study-specific procedures.

### Recombinant Fab production in *E. coli*

Heavy and Light chain variable regions of single plasma blasts, isolated from peripheral blood, were amplified and cloned into pET22 as a bicistronic expression cassette encoding for Fab antibody fragments. Fabs anti-fHbp were expressed as recombinant proteins with a C-terminal hexahistidine tag in *E. coli* Rosetta 2 (DE3). Cultures were grown in Enpresso B or human trabecular meshwork cell (HTMC) medium (autoinduced medium developed in house), and protein expression was induced by isopropyl β-D-1-thiogalactopyranoside (IPTG), 1 mM for 24 h at 25°C. Cell lysis was performed using various techniques: chemical lysis, osmotic shock, and sonication. The recombinant antibodies were purified by immobilized metal ion chromatography using nickel-nitrilotriacetic acid (Ni-NTA) agarose resin (Qiagen, Germantown, MD, USA), according to the manufacturer’s instruction. Recombinant Fabs were quantified by bicinchoninic acid assay, and their purity was assessed by SDS-PAGE after Coomassie staining in reducing and nonreducing conditions.

### fHbp variants expression in *E. coli*

fHbp variants were expressed as recombinant protein with a C-terminal hexahistidine tag in *E. coli* BL21 (DE3). Cultures were grown in HTMC medium and protein expression was induced by IPTG 1 mM for 24 h at 25°C. *E. coli* cells were lysed by cell lytic express (MilliporeSigma, Burlington, MA, USA) as in the manufacturer’s instruction and centrifuged at 9000 rpm for 30 min. The soluble fraction was then filtered by 0.22-μl filter (MilliporeSigma) to remove cell debris and purified by affinity chromatography. Protein concentration was determined by NanoDrop Spectrophotometer (Thermo Fisher Scientific, Waltham, MA, USA), and its purity was assessed by SDS-PAGE on a 4–12% Bis-Tris Gel after Problue Safe Stain (Giotto Biotech, Florence, Italy). When necessary, a second purification step of ion-exchange chromatography was performed to remove all the impurities using a prepacked HiLoad 26/60 Column Superdex 75 Prep Grade (GE Healthcare, Waukesha, WI, USA), according to the manufacturer’s protocol.

### Immunoassay by Gyrolab

All Fabs were run by Gyrolab 10 µg/ml diluted in Rexxip H. Capture reagents biotinylated fHbp v1, fHbp v2, and fHbp v3 were used at 100 µg/ml. Detection reagents goat anti-human IgG and Fab fragment specific-Alexa 647 (Jackson ImmunoResearch Laboratories, West Grove, PA, USA) were used at 25 nM. All Fabs were analyzed using Gyrolab Bioaffy 200 CDs and the standard Gyrolab 3-step method (capture-analyte-detection). The threshold for positive Fab binding was defined when the fluorescent signal was higher than 10.

### Recombinant mAbs production in mammalian cells

The genes of the variable region (V) of the heavy (H) and light (L) chains of human mAb, codon optimized for mammalian expression, were synthesized by Geneart (Thermo Fisher Scientific) by modifying the 5′ and 3′ extremities with the Eco31I site. Synthetic DNA strings were then digested with Eco31I restriction enzyme. Digested and purified DNA products were ligated into human pRS5a Igγ1, Igκ, and Igλ expression vectors containing a human Ig gene signal peptide sequence and the Eco31I cloning site upstream of the human IgG1, Igκ, or Igλ constant regions. Cloning was performed in *E. coli* strain MachI (Thermo Fisher Scientific) using standard ligation protocol. In the pRS5a antibody expression vectors, the transcription is under the cytomegalovirus promotor in frame with a human leader sequence for secretion derived by human Ig (Novartis Institutes for BioMedical Research, Cambridge, MA, USA).

The recombinant full IgGs were expressed using Expi293 (Thermo Fisher Scientific) by transient transfection in suspension, according to the manufacturer’s instructions. Cells were centrifuged at 900 rpm for 10 min 3 and 6 d after transfection, and the supernatant was collected and filtered by 0.22 μl filter (MilliporeSigma) to remove cell debris.

The recombinant IgGs were purified by affinity chromatography using a Protein G Sepharose 4 Fast Flow (GE Healthcare) according to the manufacturer’s protocol. Purified mAbs were exchanged in PBS using a PD-10 Desalting Column (GE Healthcare) and quantified using absorbance at 280 nm by NanoDrop Spectrophotometer (Thermo Fisher Scientific), and their purity was assessed by SDS-PAGE gel after Coomassie staining (Problue Safe Stain; Giotto Biotech) in reducing and nonreducing conditions.

### Recombinant Fab production in mammalian cells

For the expression of recombinant Fab, the variable regions of the heavy and light chains were cloned into a human pRS5a expression vectors encoding the Fab constant fragment with a fused C-terminal Strep-tag and expressed as previously described for mAbs production. To further purify the Fabs, the supernatant, after medium exchange with PBS, was loaded onto a StrepTrap HP Column and eluted by 2.5 mM desthiobiotin buffer (IBA solution for life). The Strep-tag was removed by tobacco etch virus (TEV) protease cleavage in a ratio of 1:100.

### Epitope mapping of anti-fHbp mAbs by protein microarray

To identify the regions of fHbp where the binding site is localized, we used a protein microarray developed in house that contains the full length of fHbp v1, v2, and v3 and several overlapping fragments covering the entire fHbp variants sequences (41). Genes were expressed in *E. coli* as either glutathione *S*-transferase – or His-tagged fusions or thioredoxin fusions purified from the cytoplasmic fraction as soluble forms, and then the recombinant antigens were spotted on nitrocellulose-coated slides.

Nonspecific binding was minimized by preincubating protein microarray slides with a blocking solution (BlockIt, ArrayIt) for 1 h. mAbs were diluted 1:50 1:2000 in BlockIt and overlaid on the protein arrays for 1 h at room temperature. AlexaFluor 647–conjugated anti-Human IgG secondary antibody (Jackson ImmunoResearch Laboratories) was added for 1 h at room temperature in the dark before proceeding with slide scanning. Fluorescence signals were detected by using a PowerScanner Confocal Laser Scanner (Tecan, Zürich, Switzerland), and the 16-bit images were generated with PowerScanner software variant 1.2 at 10 μm/pixel resolution and processed using ImaGene 9.0 software (BioDiscovery, El Segundo, CA, USA). Elaboration and analysis of image raw fluorescence intensity data was performed using in-house–developed software and R scripts. Signals were considered as positive when their mean fluorescence intensity (MFI) value was higher than 15,000, corresponding to the MFI of protein spots after detection with anti-AlexaFluor 647 pAb (Jackson ImmunoResearch Laboratories) alone, +10 sd.

### Protein crystallization and diffraction data collection and processing

To form the fHbp/Fab complex, 7.5 mg of fHbp and 5 mg of Fab were incubated overnight at 4°C and further purified by size exclusion chromatography to remove the protein in excess, using a prepacked HiLoad 26/60 Column Superdex 75 Preparation Grade (GE Healthcare). Fab/fHbp complexes concentrated at 25 mg/ml in 50 mM Tris-HCl were screened using prepacked 96 deep-well blocks commercialized by Molecular Dimensions (Newmarket, United Kingdom) using Crystal Gryphon robot (Art Robbins Instruments, Sunnyvale, CA, USA). The purified 1E6-fHbp complex (25 mg/ml) was screened using ∼500 different crystallization conditions. The largest crystals were found in 1.0 M bicine, pH 8.5 as precipitant 40% PEG 500 MME and 20% PEG 2000. Crystals were soaked in the original mother liquor supplemented with 15% ethylene glycol prior to cryo-cooling in liquid nitrogen.

### Structure determination and refinement

Diffraction of the crystals was tested at beamline ID29 of the European Synchrotron Radiation Facility, and several full data sets were collected at 100 K, at wavelength λ = 0.983 Å, on a Pilatus 6 M detector. Diffraction data sets were indexed and integrated using iMosflm and reduced using Aimless *via* the Collaborative Computational Project No. 4C(CP4) suite (43). Crystals of the fHbp-1E6 complex belong to space group P 2 2_1_ 2_1_, with the asymmetric unit containing 1 complex and a solvent content of 64.4% (Matthews coefficient of 3.57 Å^3^/Da). The structure of the complex was solved at 2.66 Å resolution by molecular replacement with Phaser (44) using as model templates for fHbp, light chain and heavy chains the Protein Data Bank (PDB; *http://www.rcsb.org/*) entries 4AYI, 3PIQ, and 5I17, respectively. The coordinates of the 1E6/fHbp v3 structure determined herein have been deposited in the PDB with accession code 6H2Y.

### Surface plasmon resonance

Surface plasmon resonance (SPR) was used to measure and compare the binding affinities of the tested mAb with the 3 variants of fHbp recombinant proteins. All SPR experiments were performed in running buffer pH 7.4 containing 10 mM Hepes, 150 mM NaCl, and 3 mM EDTA supplemented with 0.05% (v/v) P20 surfactant using Biacore T200 (GE Healthcare) at 25°C. In total, 9000–10,000 response units of an anti-huFab binder were immobilized on CM5 sensor chip, using the Human Fab Antibody Capture Kit (GE Healthcare). To determine the *K_*d*_* and kinetics parameters, schematically, the experiments were divided into 3 main steps. First, 800–1200 response units of the tested mAb at 20 μg/μl was captured by anti-Fab binder on the surface of the chip in running buffer. An anti-Fab binder coated flow cell without captured mAb was used as blank reference.

A single-cycle kinetics experiment was then performed where the fHbp analyte was injected in 5 incremental concentrations (0.39, 0.78, 1.56, 3.13, 6.25 nM) at a flow rate of 40 μl/min.

Finally, the chip surfaces were regenerated using a buffer containing 10 mM glycine pH 2.1 (180 s, flow rate 10 μl/min). Each experiment was performed in duplicate.

A blank injection of buffer only was subtracted from each curve, and reference sensorgrams were subtracted from experimental sensorgrams to yield curves representing specific binding. SPR data were analyzed using the Biacore T200 Evaluation software (GE Healthcare). Each sensogram was fitted with the 1:1 Langmuir binding model, including a term to account for potential mass transfer, to obtain the individual *k*_on_ and *k*_off_ kinetic constants; the individual values were then combined to derive the single averaged *K*_d_ values reported.

### Serum bactericidal activity assay

Serum bactericidal activity (SBA) against *N. meningitidis* strains was evaluated as reported by McCoy *et al.* (45). Bacteria grown until early log phase [optical density (OD)_600_ of ∼0.25] were diluted in Dulbecco PBS (D8662; MilliporeSigma) containing 1% bovine serum albumin (BSA) and 0.1% glucose at the working dilution of 10^4^–10^5^ and incubated with serial 2-fold dilutions of test mAb starting from a concentration of 125 µg/ml. Serum bactericidal titers were defined as the mAb dilution, resulting in a 50% decrease in colony forming units (CFU) per milliliter after a 60-min incubation of bacteria with the reaction mixture compared to the control CFU per milliliter at time 0. Pooled baby rabbit sera from Cedarlane or human serum, obtained from volunteer donors under informed consent, have been used as a complement source for rabbit or human SBA, respectively.

### Inhibition of binding of hfH

The ability of the mAb to inhibit binding of factor H (fH) to live bacteria was measured by flow cytometry. Bacterial cells grown until midlog phase (OD_600_ of ∼0.5) were incubated with anti-fHbp mAb (50 μg/ml for UK320, 10 μg/ml for MC58, and UK104 in PBS-1% BSA buffer) for 30 min at room temperature, followed by the addition of purified human fH (25 μg/ml for UK320, 50 μg/ml for MC58 and UK104), which was incubated for an additional 30 min at room temperature in a final reaction volume of 100 μl. fH binding was detected with a goat polyclonal antiserum to fH (341276; MilliporeSigma) diluted 1:100 and incubated for 30 min at room temperature, followed by additional 30 min incubation with a donkey anti-goat IgG fluoresceinisothiocyanate conjugate (705.095.003; Jackson ImmunoResearch Laboratories) diluted 1:100 in PBS-1% BSA buffer.

### Data availability

All data generated or analyzed during this study were included in this published article (and in the Supplemental Data). The data sets generated during protein chip and PepScan and were analyzed and are available in the Gene Expression Omnibus database (*http://www.ncbi.nlm.nih.gov/geo/query/acc.cgi*) under series accession no. GSE98883.

## RESULTS

### Thirteen crossreactive huFabs targeting fHbp identified

**Figure 1:**
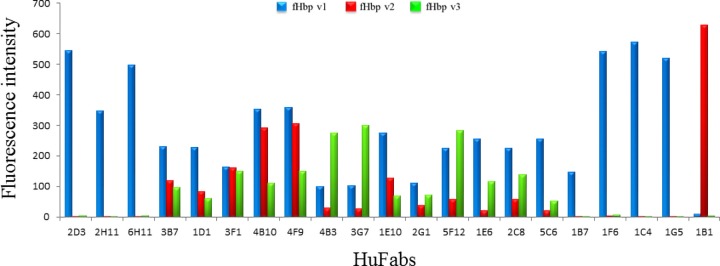
Thirteen crossreactive anti-fHbp huFabs were identified. Histograms represent the binding specificity of a subset of 21 of the 110 huFabs analyzed by Gyros immunobinding assay; all the crossreactive Fabs identified have been included in this figure. The blue, red, and green bars refer to the binding of a specific Fab (labeled beneath) to fHbp v1, v2, and v3, respectively.

To investigate the anti-fHbp repertoire of 3 adults immunized with 4CMenB, the variable region of the IgG heavy chains (VH) and variable region of the IgG light chains (VL) were isolated from peripheral blood mononuclear cells and cloned into an *E. coli* expression construct as previously described by Beernink *et al.* (42), obtaining a library of 110 anti-fHbp huFab-expressing plasmids. To determine the fHbp variant binding specificity of each huFab, we set up a protocol (Supplemental Data S1) suitable to produce the recombinant 110 anti-fHbp huFabs in *E. coli* in a small scale suitable for rapid and efficient screening. After miniaturized immobilized metal affinity chromatography purification, all huFabs were analyzed using a nanoliter-scale immunoassay system (Gyros Protein Technologies, Uppsala, Sweden), a highly sensitive platform for screening crossreactivity in a qualitative way even for samples with suboptimal purity. As expected, the vast majority of the 110 huFabs tested recognized the fHbp variant 1 (Supplemental Figs. S2 and S3). Moreover, although only the fHbp v1 is included in the 4CMenB vaccine, 13 huFabs resulted to be crossreactive between the 3 main variants of fHbp (**Fig. 1**). The latter finding was of particular interest, because previously only 2 anti-fHbp huFabs were reported to be crossreactive (41).

### Protein microarray epitope mapping suggests mAbs have different binding profiles

To map and identify the epitope binding regions and to further confirm crossreactivity with different fHbp variants, each antibody of the 13 crossreactive Fabs subset was produced in mammalian cells as recombinant full-length IgG1 because this is the most abundant Ig subclass in human sera (46). The recombinant mAbs were then tested in a protein microarray containing full-length and different overlapping recombinant fragments covering the entire length of the fHbp v1, v2, and v3. The data of this analysis, summarized in **Table 1**, suggest that all mAbs recognize conformational epitopes on the full-length fHbp variants, except for mAb 5C6, which recognized only v1. Three mAbs (4F9, 4B3, 3G7) react with the isolated C-terminal β-barrel domain (in addition to the full-length protein), suggesting that their epitope could be localized in that fHbp region. The mAb 5F12 is unique in being able to bind the isolated N-terminal domain of the fHbp variants. The other mAbs were neither able to bind the separate N- or C-terminal domains of fHbp nor to smaller fragments.

**TABLE 1:** Crossreactivity of 13 huFabs with different fHbp variants assessed by protein microarrays containing recombinant full-length proteins and subdomains of fHbp v1, v2, and v3

mAbs	fHbp v1			fHbp v2			fHbp v3		
	Full length	C-term β-barrel domain	N-terminal domain	Full length	β-Barrel	N-terminal	Full length	β-Barrel	N-terminal
3B7	X			X			X		
1D1	X			X			X		
3F1	X			X			X		
4B10	X			X			X		
1E10	X			X			X		
2G1	X			X			X		
5F12	X		X	X		X	X		X
1E6	X			X			X		
2C8	X			X			X		
5C6	X								
4F9	X	X		X	X		X	X	
4B3	X	X		X	X		X	X	
3G7	X	X		X	X		X	X	

“X” indicates which regions of fHbp were bound by the mAb, whereas empty cells indicate that no signal was detected.

Protein microarray data, including the data regarding the smaller fragments and domains that are not recognized and are not reported in Table 1, have been deposited in the National Center for Biotechnology Information Gene Expression Omnibus database (*http://www.ncbi.nlm.nih.gov/geo/query/acc.cgi*).

### Crystal structure of huFab 1E6 in complex with fHbp v.3

To obtain detailed information about the conserved epitopes in the fHbp variants, a subset of the 13 mAbs was selected for further structural studies and produced as Fabs in mammalian cells. The crystal structure of huFab 1E6 in complex with fHbp v.3 was determined at 2.7 Å resolution. X-ray data collection, processing, and refinement statistics are shown in **Table 2**. The structure was solved by the molecular replacement method, and the resulting electron density maps allowed unambiguous model building.

**TABLE 2:** Data collection and refinement statistics

Variable	fHbp (v3): 1E6 complex (PDB code 6H2Y)
Crystal	
* *Space group	P22_1_2_1_
Cell dimensions	
* a*, *b*, *c* (Å)	42.97, 65.27, 278.82
* *β (deg)	90.00
Data collection	
* *Beamline	ESRF ID29
* *Wavelength (Å)	0.983
* *Resolution (Å)	63.56–2.64 (2.74–2.64)
* *Total reflections	93,094 (14,071)
* *Unique reflections	23,201 (3370)
* R*_merge_	0.159 (0.56)
* R*_meas_	0.180 (0.637)
* I/*σ(*I*)	9.5 (2.4)
* CC*_1/2_	0.987 (0.472)
* *Completeness (%)	97.7 (98.7)
* *Redundancy	4.0 (4.2)
* *Wilson *B* factor (Å)	32.90
Refinement	
* *Resolution (Å)	63.56–2.65
* *No. reflections	23,182
* R*_work_/*R*_free_	20.46/27.18
No. atoms	
* *Protein	5131
* *Ligand/ion	16
* *Water	75
*B* factors	
* *Protein	43.92
* *Ligand/ion	41.41
* *Water	36.27
Root-mean-square deviations	
* *Bond lengths (Å)	0.01
* *Bond angles (deg)	1.44
* *Clash scores	11.66
Ramachandran	
* *Favored (%)	91.77
* *Allowed (%)	7.4

*R*_sym_ = Σ *hkl* Σ *i jIi(hkl)* − *〈 I(hkl) 〉 j*/Σ *hkl* Σ i *Ii(hkl)*. *R*_work_ = Σ *jjF*_obs_*j* – *jF*_calc_*jj* / Σ *jF*_obs_*j*. *R*_free_ is the same as for *R*_work_ but calculated for 5% of the total reflections that were chosen at random and omitted from refinement. ESRF, European Synchrotron Radiation Facility.

Values in parentheses are for highest-resolution shell.

**Figure 2:**
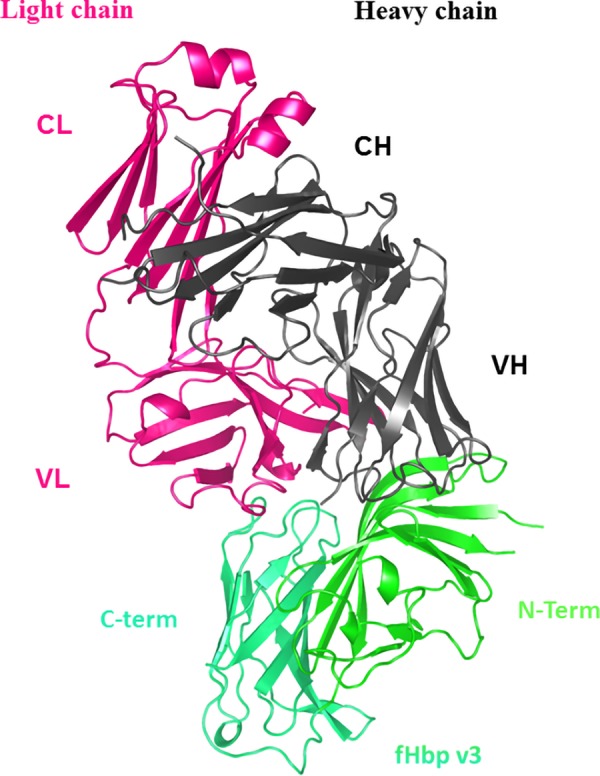
HuFab 1E6 in complex with fHbp v3. The crystal structure of fHbp v3 (green) and huFab 1E6 are shown, with the heavy chain (CH and VH) and the light chain (CL and VL) in black and pink, respectively.

Considered alone, the 2 proteins maintained their typical structural characteristics (**Fig. 2**); the fHbp assumed the canonical 3D structure of 2 β-barrels connected by a short linker, and the Fab exhibits the standard Ig domain fold (47, 48). The structure of the 1E6 Ig domains consists of antiparallel β-sheets arranged in a sandwich fashion, with the heavy chain folding into VH and CH domains and the light chain folding into VL and CL domains. The 2 sides of the sandwich motif are covalently linked by disulfide bonds. The elbow angle, defined by the relative displacement of the variable domains and the constant domains, is 138.2° (49).

**Figure 3:**
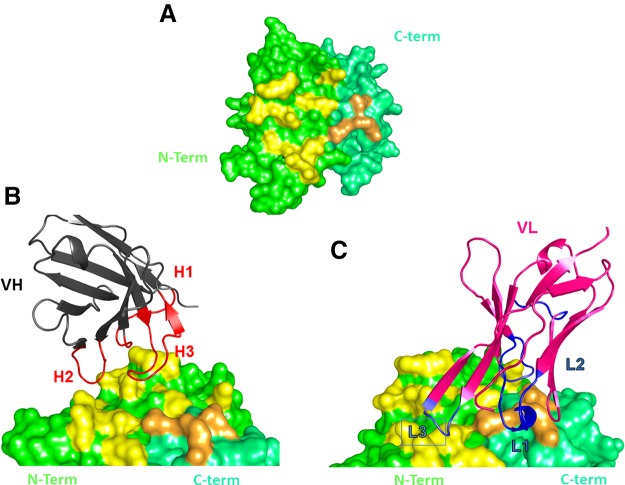
Surface representation highlighting the 1E6 epitope. *A*) View of the 1E6 epitope on the surface of fHbp V3. Residues located at the N-terminal domain are colored yellow, whereas those in the C-terminal β-barrel domain are orange. *B*, *C*) Residues targeted by the heavy chain and by the light chain are shown. Variable region of heavy (black, *B*) and light chain (pink, *C*) are represented, the H-CDR are highlighted in red and the L-CDR in blue.

The complementarity determining regions (CDRs) L1, L2, H1, and H2 of huFab 1E6 assumed canonical structures similar to Chothia class 2, 1, 1, 2 (50). The CDR-L3 shows a noncanonical conformation with standard loop length. HuFab 1E6 forms a broad network of interactions with the N-terminal region of the fHbp v.3, whereas only 3 residues of the C-terminal domain are involved in the binding. The calculated interface buries a total area of 1058 Å^2^, which is in the typical range of the interaction surface between antibodies and Fab:antigens (51, 52), with the N-terminal β-barrel mainly involved in the interaction (**Fig. 3*A***). Both chains of huFab 1E6 contribute to binding of the fHbp v.3 with the heavy chain that contacts only the N-terminal domain defining an interface area of 551 Å^2^, whereas the light chain contacts both domains of fHbp (Fig. 3*B, C*) on a surface of 507 Å^2^. The binding does not follow the classic “lock and key or induced fit model” (53); indeed, the epitope is localized on a rather flat area (Fig. 3*A*). Nevertheless, several polar and electrostatic interactions are formed, mediated by side chain atoms from all 6 CDRs (**Table 3**).

**TABLE 3:** Molecular interactions between key residues in the Fab 1E6:fHbp v3 epitope

fHbp v3	Fab 1E6	CDR	Bond type
S53	L54	H2	VdW
Q55	Y32, D100	H1, H3	Hydrogen bond
G56	D31, Y32	H1, H1	VdW, VdW
E58	R56	H2	Salt bridge
K79	D52	L2	Salt bridge, VdW
R82	T101	H3	Hydrogen bond
D84	T101, A102	H3	Salt bridge, salt bridge
V86	L54	H2	VdW
S100	F55	H2	VdW
E119	R92	L3	Salt bridge
K120	D95	L3	Salt bridge
N132	D95	L3	Hydrogen bond
D166	T31, G29, R92,	L1, L1, L3	VdW, hydrogen bond, salt bridge
N169	S51, D50	L2, L2	Salt bridge, hydrogen bond
K191	D50, A28, K30	L2, L1, L1	Salt bridge, hydrogen bond, hydrogen bond

VdW, Van der Waals.

**Figure 4:**
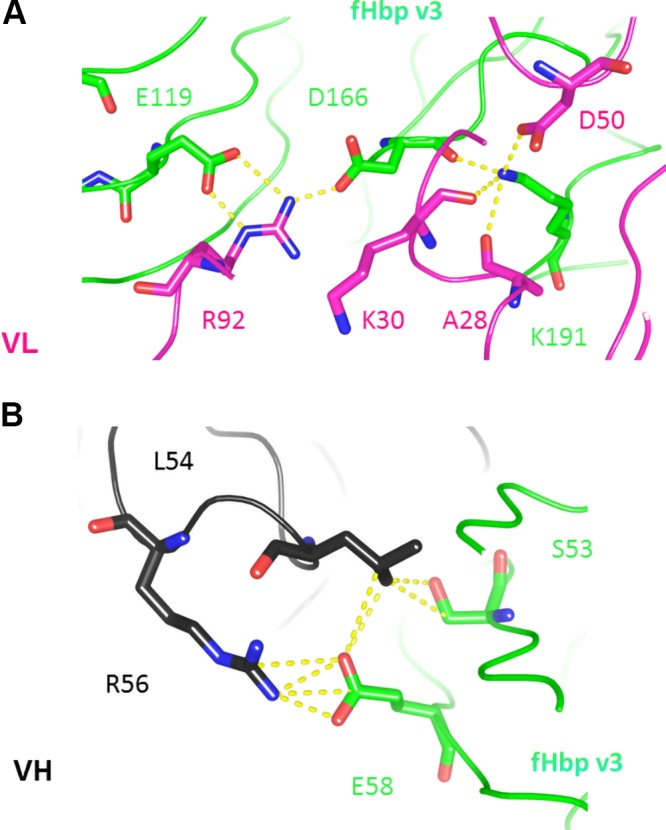
A closer view of key residues (shown as sticks) between N-terminal of fHbp v3 in green and the light chain in pink (*A*) and the heavy chain in black (*B*). In both panels, yellow dashed lines indicate intermolecular H bonds or salt bridges <4 Å.

X-ray structures of the complex between antigens and antibodies are essential to clearly define the details of epitope:paratope interactions (54). Closer examination of the key residues revealed many of polar nature, forming hydrogen bonds and salt bridges, which contribute to the binding of this mAb to the meningococcal fHbp. Notably, some residues engaged a complex network of molecular interactions that connected more than 2 aa, such as the interconnection established between fHbp v3 E119, D166, K191, and the light chain A28, K30, D50, R92 (**Fig. 4*A***), and fHbp v.3 residues E58, S53, and heavy chain L54, R56 (Fig. 4*B*).

### High conservation of the epitope underlies the broad crossreactivity of mAb 1E6

**Figure 5:**
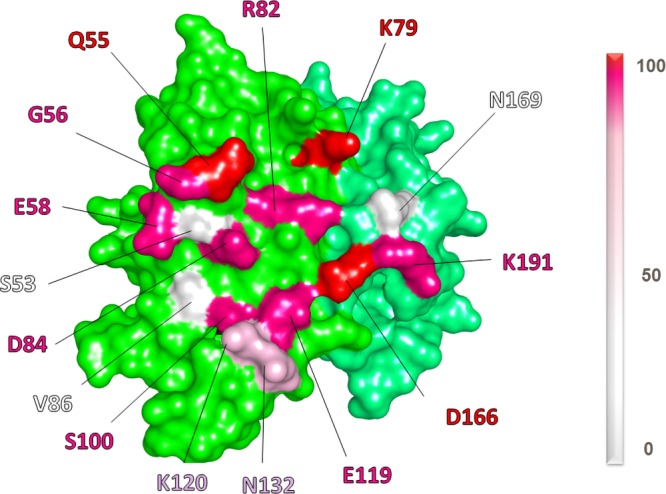
Allelic conservation of the key residues of fHbp v3 involved in the interaction with Fab 1E6. The figure shows fHbp v3 as solid surface. Each residue of the epitope is colored based on its conservation among the fHbp gene repertoire, which included 1119 allelic sequences.

Antigenic drift is the continuous process of genetic and antigenic changes that occur through point mutations. The antigenic distance between meningococcal strains increases with time as the drift increases the grade of variability to escape the host immune system. Several studies of the diversity of the fHbp gene and the encoded protein in a representative sample of meningococcal isolates confirmed high variability in this antigen, with sequence identities falling as low as ∼62% for some pairwise comparisons (48, 55). Therefore, to qualitatively estimate the extent of crossreactivity of the mAb 1E6, we calculated the percentage of conservation of the 15 fHbp residues within the epitope. A total of 1119 different fHbp alleles were retrieved from the public database *N. meningitidis* multilocus sequence typing (55), and the degree of conservation of each epitope residue was noted (**Fig. 5** and Supplemental Table S1). Interestingly, 10 out of 15 residues are conserved in >99% between all currently known fHbp amino acid sequences. Remarkable instances are represented by Q55, K79, D166 present in 100% of known meningococcal fHbp sequences. Those 3 epitope residues play key roles in binding to mAb 1E6. Of the remaining 5 residues comprising the epitope, K120 and N132 are conserved in 40% of the isolates, whereas the S53, V86, and N169 are represented in <30% of meningococcal strains (Fig. 5 and Supplemental Table S1). Collectively, these observations suggest that mAb 1E6, shown to bind representatives of the 3 fHbp variant groups, might indeed display broad crossreactivity across the entire repertoire of fHbp.

### Human mAb 1E6 binds fHbp in a different region compared to mAb 1A12 and on the opposite side of hfH binding site

**Figure 6:**
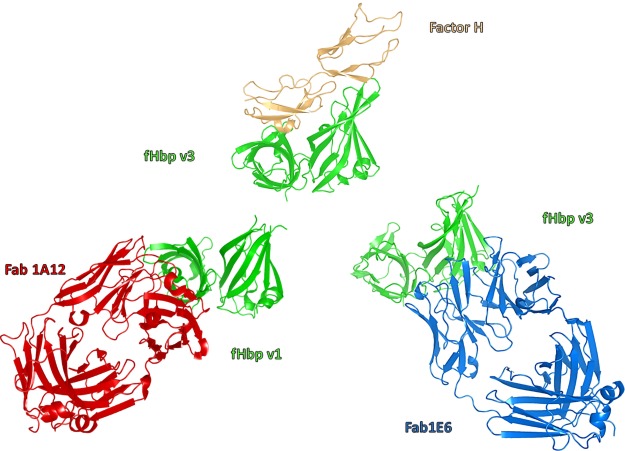
Cartoon representations of fHbp (green), bound to Fab 1A12 (red), Fab 1E6 (blue), and hfH (orange). fHbp is shown in the same orientation in all panels.

To date, several human anti-fHbp antibodies have been described in functional assays (41, 42, 56); however, to our knowledge, only 2 examples of epitope mapping at the atomic level of anti-fHbp antibodies elicited in humans have been reported so far. Giuliani *et al.* (41) restricted the epitope localization of 1 crossreactive anti-fHbp mAb 1G3 to short fragments of fHbp v1 by hydrogen-deuterium exchange–MS, whereas López-Sagaseta *et al.* (18) were able to fully characterize at high resolution, by X-ray crystallography, the first human antibody, the 1A12, identifying the epitope on fHbp v1. Fab 1A12 targets exclusively the C-terminal β-barrel, whereas the Fab 1E6 mainly binds the N-terminal region on the opposite side compared to the binding site for hfH (**Fig. 6**). The latter suggested that Fab 1E6 would not inhibit the binding of fHbp to hfH. This hypothesis was confirmed using a competition assay performed by flow cytometry (Supplemental Fig. S4). Three residues of the C-terminal β-barrel of fHbp v.3 are involved in 1E6 binding, but only residue K191 engaged a strong network of interactions results to be a crucial residue in both fHbp v1:Fab 1A12 and fHbp v3:Fab 1E6 complexes, further underlining the important antigenic role of this specific amino acid.

### Human mAb 1E6 binds tightly to all 3 fHbp variants

**Figure 7:**
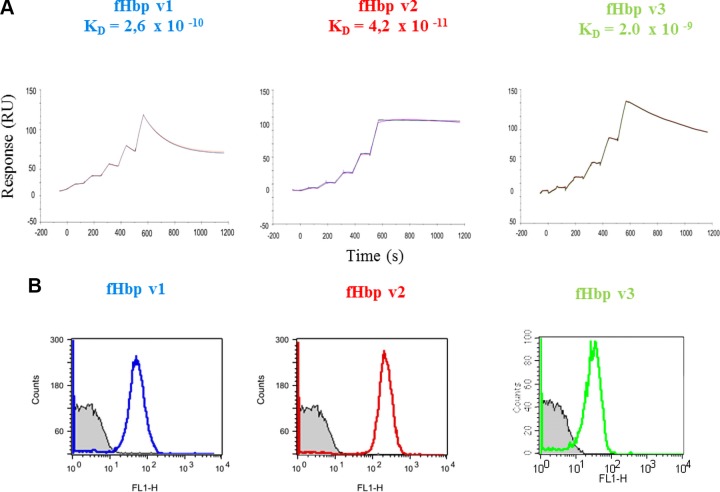
*A*) Histograms showing binding to live meningococci of MenB strains when incubated with 10 μg/ml of HumAbs anti-fHbp (blue line, MC58 strain; red line UK104 strain; green line, UK320 strain). Gray-filled histogram represents negative control, bacteria incubated with PBS and anti-human IgG fluoresceinisothiocyanate conjugated. *B*) Sensorgrams show the experimental association and dissociation traces (colored) of binding of mAb 1E6 on 5 incremental concentrations of fHbp v1, v2, and v3, respectively, performed in duplicate. The calculated fitting traces are shown in black.

To connect structure with function, we decided to perform a deeper biochemical characterization of this antibody. We performed SPR analyses to investigate the interaction between mAb 1E6 and the purified recombinant fHbp variants in terms of binding affinity and association/dissociation kinetics (**Fig. 7*A***). Although the vaccine contains only fHbp v1, the analysis confirmed that mAb 1E6 recognized all the 3 main variants of fHbp with high affinity, with *K*_d_ values in the low nanomolar range for v3, and even tighter for v1 and v2 (**Table 4**). Kinetic constants measured suggest a different degree of stability of the complexes when mAb 1E6 bound different variants of fHbp (Table 4 and Fig. 7*A*). The most stable binding was measured on fHbp v2 because mAb 1E6 seems to associate rapidly and form a very stable complex with slow dissociation. On the other hand, the affinity for the v1 is 10-fold lower than with v2, and the rapid association of the mAb to fHbp followed by a fast dissociation suggests the formation of a shorter lived 1E6/fHbp v1 complex.

**TABLE 4:** Kinetic constants measured by SPR

Type	fHbp v1	fHbp v2	fHbp v3
*K*_a_ (1/Ms)	5.25E+7 ± 0.15	1.2E+5 ± 0.15	5.7E+5 ± 1.8
*K*_d_ (1/s)	1.4E−2 ± 0.01	4.3E−4 ± 1.3	1.3E−3 ± 0.3
*K*_d_ (M)	2.65E−10 ± 0.15	3.6E−11 ± 0.7	2.7E−9 ± 1.3

*K*_d_ = *k*_off/_*K*_on_; mean ± sd values were calculated from SPR experiments performed in duplicate for each fHbp variant and mutant. Binding affinity and association/dissociation kinetics were measured at Biacore T200.

### Human mAb 1E6 recognizes native fHbp on live bacteria

Meningococcal fHbp is a surface-exposed lipoprotein bound to the outer membrane by an N-terminal lipid anchor. Therefore, flow cytometry is a suitable tool to detect the anti-fHbp mAbs binding directly to the surface of the bacteria. Fluorescence-activated cell sorting analysis confirmed that human mAb 1E6 is crossreactive because it is able to bind different variants of fHbp on the surface of live meningococcal strains MC58, M08-0240104 (UK104), and M01-0240320 (UK320), which express fHbp v1, v2, and v3, respectively. We also observed that the fluorescent signal was 10-fold stronger on the meningococcal strain expressing v2 compared to v1 and v3 (Fig. 7*A*). The amount of fHbp present on the bacterial surface varies between meningococcal strains, and it has been previously described by Biagini *et al.* (57) that the strain M08-0240104 displays an fHbp surface density higher than the other strains, likely explaining the higher fluorescence intensity measured.

### The 1E6 mAb is crossbactericidal in presence of rabbit complement

The bactericidal activity of the human mAb 1E6 was tested against MenB strains expressing distinct fHbp v1, v2, and v3 using baby rabbit and human serum as source of exogenous complement. SBA is a functional serological surrogate of vaccine efficacy (14, 58, 59). When tested in human SBA (with human complement source) against strains carrying 1 of the 3 fHbp variants, the mAb 1E6 alone failed in eliciting positive titers (<4). In contrast, when baby rabbit serum was used as the source of exogenous complement, the mAb 1E6 showed positive bactericidal titers against meningococcal strains carrying different fHbp variants. However, the extent of functionality varied between strains; the higher titers were measured against the M08-0240104 strain expressing the fHbp v2, rabbit serum bactericidal assay (rSBA) titer value of 4096, compared to lower rSBA titers of 256 and 128 when tested against M11295 expressing the fHbp v1 or UK320 expressing the fHbp v3, respectively. Notably, rSBA titers correlated with the affinity of mAb 1E6 for the recombinant proteins measured herein and with the previously reported surface density of the antigens (57).

## DISCUSSION

*N. meningitidis* remains the most common cause of bacterial meningitis, often leading to permanent disabilities or even death (60). After many years of research and development, 2 vaccines to protect against MenB disease are now licensed, and both contain fHbp, a key meningococcal virulence factor. Recent data from the United Kingdom infant immunization campaign against MenB reported >80% 4CMenB vaccine-mediated protection (61). Here, we report detailed molecular studies that describe important elements of the human antibody response to 4CMenB.

As an extension of previous smaller-scale investigations (41, 42), the study presented here probes the repertoire of human mAbs isolated for a long time from 3 vaccine recipients following vaccine administration. From this study, a new library of huFab expression constructs was obtained. The library encodes over 100 distinct huFabs that recognize fHbp. Most notably, even though the vaccine contains specifically the fHbp variant 1, subjects immunized with 4CMenB produced at least some crossreactive antibodies, and we identified 13 (of 110 total) huFabs that were able to recognize all 3 fHbp variants (Fig. 1). Protein microarray epitope mapping experiments suggested that these 13 crossreactive huFabs bind diverse regions of fHbp, mostly involving conformational epitopes requiring the full-length fHbp, although 3 huFabs bind to the isolated C-terminal β-barrel domain and, remarkably, just 1 huFab binds to the isolated N-terminal domain (Table 1).

To date, the 3D structure of only 1 huFab (1A12) in complex with fHbp variant 1 has been reported (18). Here, we present the crystal structure of fHbp variant 3 (only 58% sequence identify with variant 1) in complex with a quite different crossreactive huFab, 1E6 to deepen our understanding of the molecular bases underlying the crossreactive antibody response in humans (Fig. 2). The structure revealed a large conformational epitope, featuring a dense network of salt bridges and hydrogen bonds, mainly localized at the N terminus of fHbp variant 3 that was not previously seen in other crystal structures of fHbp complexed with murine Fabs, nor with huFab 1A12, which targets a very different region on the C-terminal domain of fHbp (Figs. 3–6).

In biochemical SPR studies, huFab 1E6 was found to bind tightly to fHbp variants 1, 2, and 3, with highest affinity for variant 2 (Fig. 7*A*). Similarly, huFab 1E6 bound to live meningococcal cells expressing fHbp variant 1, 2, and 3 when examined by flow cytometry (Fig. 7*B*). The crystal structure enabled a clear understanding of this crossreactivity. Of 15 total fHbp variant 3 residues involved in binding to huFab 1E6 (Fig. 5), 10 are fully conserved in the variants 1 and 2 tested herein (Supplemental Fig. S5). Further, we calculated the degree of conservation of epitope residues considering the 1119 fHbp alleles (from clinical isolates and carrier strains) deposited in the Bacterial Isolate Genome Sequence Database (*https://bigsdb.readthedocs.io/en/latest/*) (55). Again, we found out that 10 of the 15 epitope residues are conserved >99% in the entire known fHbp sequence database, suggesting that mAb 1E6 could have a very broad recognition of most circulating meningococcal strains.

A similar relationship between kinetic binding parameters measured in an *in vitro* SPR assay were also found in an *in vivo* assay measuring binding of 1E6 to fHbp naturally exposed on MenB strains and in a bactericidal activity assay. Antibodies have 2 distinct functions: one is to bind specifically to their target antigens, and the other is to elicit an immune response recruiting other cells and molecules. Previous studies demonstrated that several factors contribute to bactericidal activity in the presence of human serum of anti-fHbp mAbs, such as human IgG subclass, alternative complement pathway activation, and epitope density (29–31). However, anti-fHbp mAbs that individually did not elicit bactericidal activity could became bactericidal when mixed (36, 41, 42). We found that 1E6 can efficiently activate the complement cascade in a rabbit serum bactericidal assay (rSBA), but is not able alone to induce human anti-FHbp bactericidal activity. A likely explanation is that fHbp present on the bacterial surface binds to hfH (binding of fH to fHbp is human-specific), which protects bacteria from complement-mediated killing. Another possible explanation is species-specific differences in C1q engagement by 1E6. Our results are consistent with prior studies with anti-fHbp mAbs (murine or human), where human complement-mediated bactericidal activity was observed only if the mAb blocked fH-binding or was used in combination with other mAbs (29, 30, 41). Previous studies demonstrated that the quantity of fHbp present on the bacterial surface varies between isolates and in the strains used, MC58, M08-0240104, and UK320 the concentration of fHbp was determined to be 2900 (fHbp v1), 9390 (fHbp v2), and 1111(fHbp v3) molecules for cells, respectively (57). The 9-fold higher antigen density displayed on the surface of strain M08-0240104 explains why the binding on v2 is higher than on v1 and v3 (Fig. 7*B*). The ability of meningococci to selectively bind to hfH enable the bacteria to be more resistant to the immune system in 2 ways: directly down-regulating the complement alternative pathway or preventing the antibodies competitively binding for the same hfH binding site on fHbp, or both (13). The crystal structure of fHbp v3 in complex with the complement control protein domain 6 and 7 of hfH was previously determined (17). Comparing the binding site of hfH and Fab 1E6, we observe that 1E6 binds fHbp v3 on the opposite side, and accordingly, the data obtained herein by flow cytometry analyses confirm that this antibody is not able to prevent the hfH binding to fHbp (Supplemental Fig. S4*A*), and the hfH cannot block the 1E6 binding to bacteria (Supplemental Fig. S4*B*).

In addition to its antigenic crossreactivity, the functional characterization of mAb 1E6 showed that it is crossprotective in rSBA. This finding suggests that vaccination with 4CMenB can induce the production of at least a small set of crossprotective antibodies in humans. In summary, here we report the identification of a highly conserved epitope, the first epitope recognized by a human mAb and predominantly localized on the N terminus of fHbp. Improving the knowledge of the epitope profiles identified by potent human antibodies could facilitate antigen engineering aiming to induce the immune system to continuously generate novel antibodies able to recognize multiple sites for a broadly protective response. Moreover, epitope mapping is a crucial step in the development of therapeutic mAbs, allowing improvements of the affinity, recognition breadth, and bactericidal efficacy potentially for treatment of meningococcal infections. The results presented here reinforce the proof-of-concept for the use of memory B-cell–derived huFab sequences in the structural and functional analysis of the human immune response after 4CMenB vaccination. This information will be useful for future vaccine research projects and to train and potentiate capabilities of emerging computational methods for antibody modeling and B-cell epitope predictions.

## Supplementary Material

This article includes supplemental data. Please visit *http://www.fasebj.org* to obtain this information.

## Abbreviations

3D: three dimensional
4CMenB: 4 component meningococcus B vaccine
BSA: bovine serum albumin
CDR: complementarity determining region
fH: factor H
fHbp: factor H binding protein
hfH: human factor H
huFab: human Fab
MenB: meningococcal serogroup B
PDB: Protein Data Bank
rSBA: rabbit serum bactericidal assay
SBA: serum bactericidal activity
SPR: surface plasmon resonance
VH: variable region of the IgG heavy chain
VL: variable region of the IgG light chain
