# Supplementary material for: Cocrystal structure of meningococcal factor H binding protein variant 3 reveals a new crossprotective epitope recognized by human mAb 1E6
Source: FASEB J. 2019 Oct 5;33(11):12099–111. doi: 10.1096/fj.201900374R (PMC6902690; doi:10.1096/fj.201900374R)
Supplement: Supplementary file 1 [file fj.201900374R.sd1.docx]

**Supplemental Data**

**Figure S1** A comparison between the two expression methods tested for high throughput Fab production. Here we reported, as example three of the 24 Fabs used in the pilot experiment, the SDS-PAGE gel of the samples run in reducing condition followed by Coomassie staining (safe blue Giotto Biotech) of the fraction from whole cell lysis by cell lytic express(a) and from periplasmic samples obtained by shock osmotic (b). MW: molecular weight.


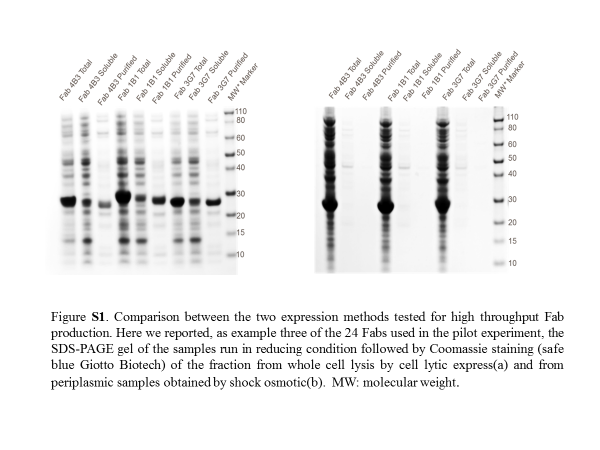


Figure S2


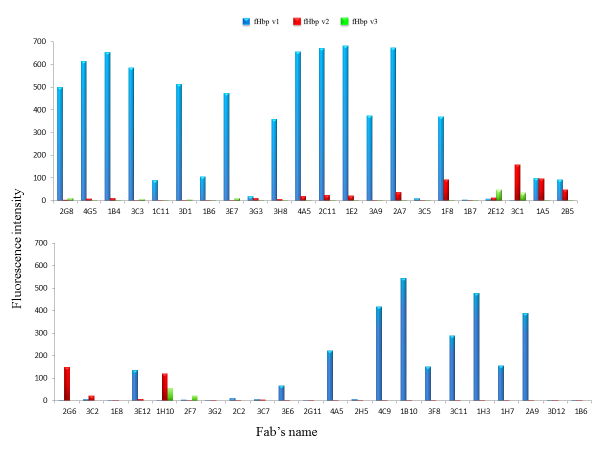


Figure S3


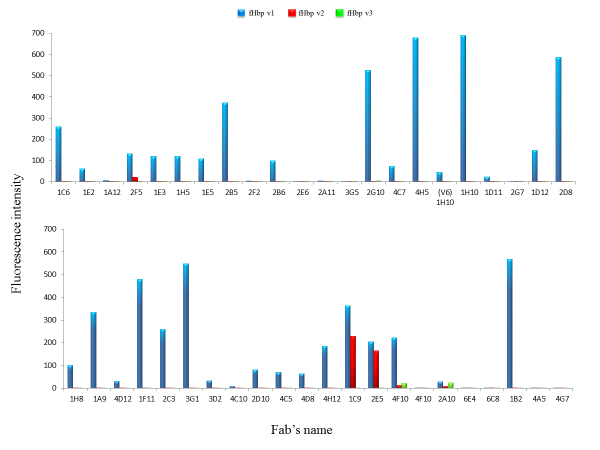


Figure S4

a)


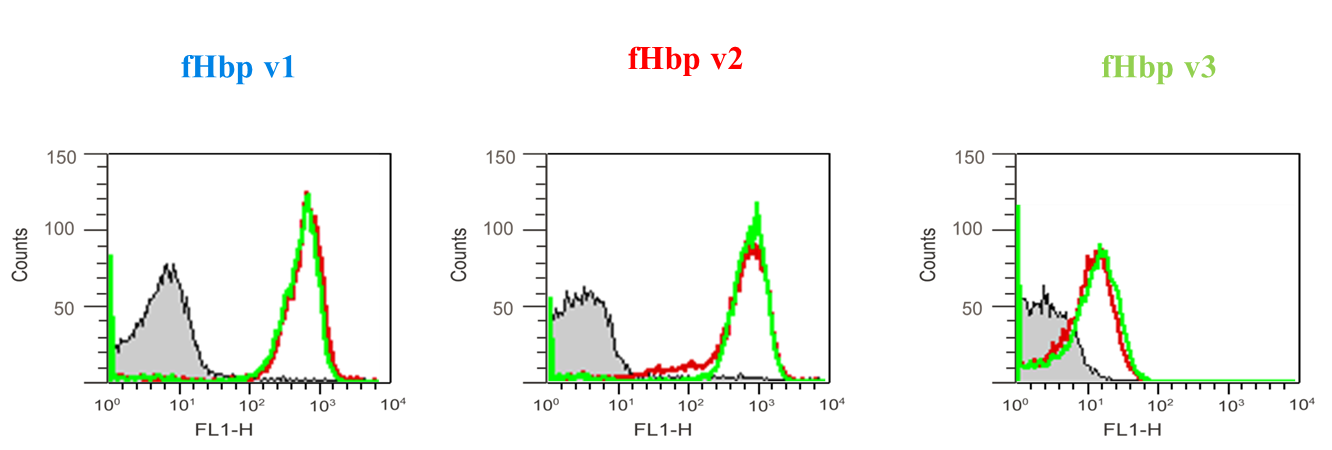


**
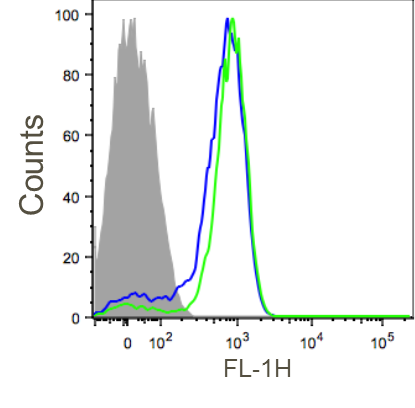
b)**

**Figure S4.** (a) Flow cytometry binding of fH on bacteria pre-incubated with PBS (red line) and pre-incubated with mAb (green line). Gray-filled area represents negative control bacteria incubated with PBS and secondary FITC-conjugated antibodies. The presence of the mAb 1E6 did not affect the binding of hfH to meningococcal strains carrying fHbp v1, fHbp v2 or fHbp v3, suggesting that hfH and mAb 1E6 target fHbp in different and distant areas. (b) Flow cytometry binding of mAb 1E6 on bacteria pre-incubated with PBS (blue line) or pre-incubated with hfH (green line).

Figure S5


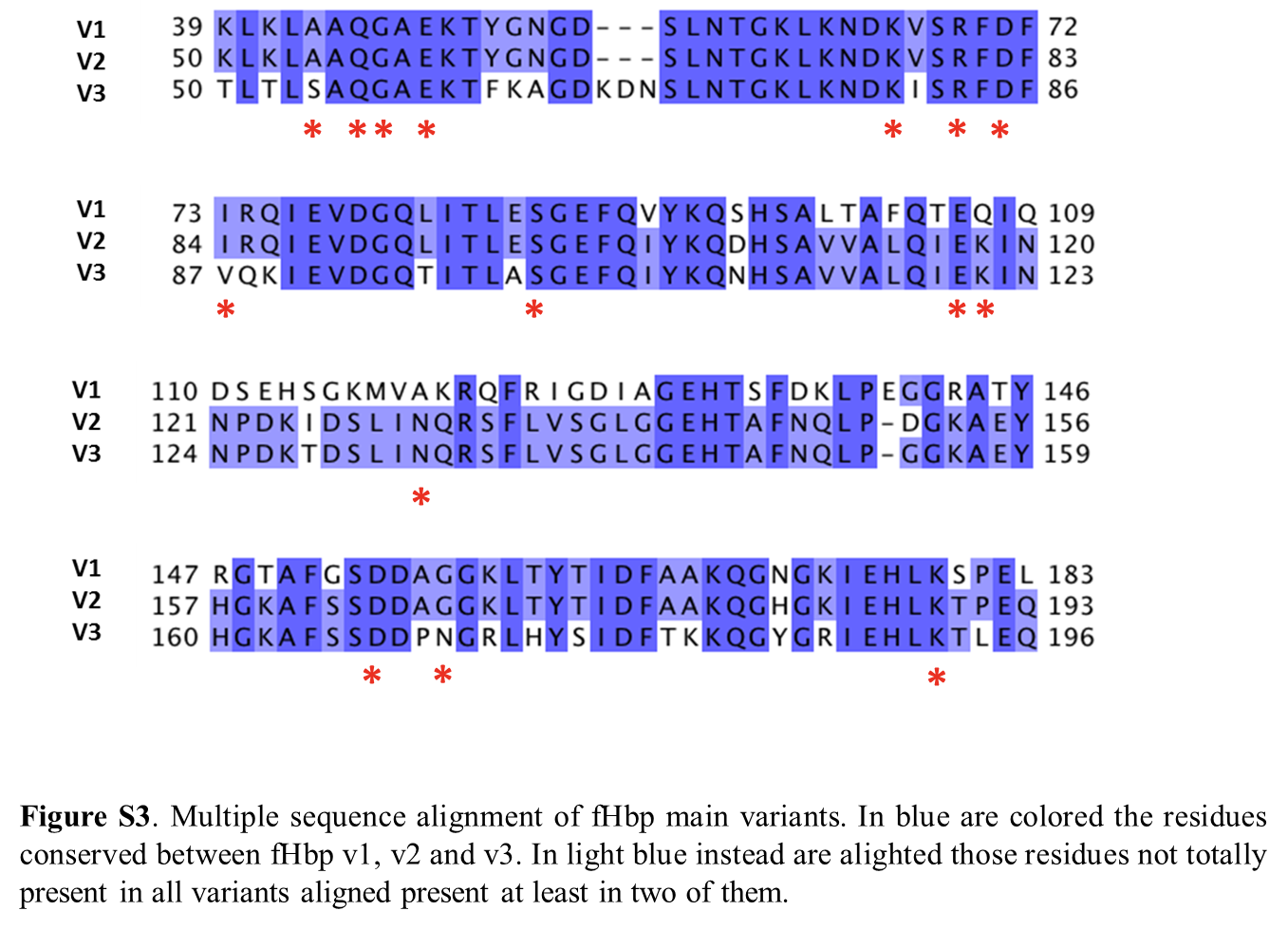


**Figure S5.** Multiple sequence alignment of fHbp main variants. In blue are colored the residues conserved between fHbp v1, v2 and v3. In light blue instead are highlighted those residues not totally present in all variants aligned, but present at least in two of them.

Table S1. Degree of conservation of the key residues involved in fHbp v3 and Ab 1E6

|  | **%G** | **%A** | **%L** | **%M** | **%F** | **%W** | **%K** | **%Q** | **%E** | **%S** | **%P** | **%V** | **%I** | **%C** | **%Y** | **%H** | **%R** | **%N** | **%D** | **%T** |
| --- | --- | --- | --- | --- | --- | --- | --- | --- | --- | --- | --- | --- | --- | --- | --- | --- | --- | --- | --- | --- |
| **S53** |  | 70.42 |  |  |  |  |  |  |  | 29.31 |  | 0.27 |  |  |  |  |  |  |  |  |
| **Q55** |  |  |  |  |  |  |  | 100 |  |  |  |  |  |  |  |  |  |  |  |  |
| **G56** | 99.82 |  |  |  |  |  |  |  | 0.09 |  |  |  |  |  |  |  | 0.09 |  |  |  |
| **E57** |  |  |  |  |  |  | 0.09 |  | 99.91 |  |  |  |  |  |  |  |  |  |  |  |
| **K79** |  |  |  |  |  |  | 100 |  |  |  |  |  |  |  |  |  |  |  |  |  |
| **R82** |  |  |  |  |  |  |  |  |  |  |  |  |  |  |  | 0.18 | 99.73 |  | 0.09 |  |
| **D84** |  |  |  |  |  |  |  |  |  |  |  |  |  |  |  |  |  | 0.18 | 99.82 |  |
| **V86** |  |  |  |  |  |  |  |  |  |  |  | 21.09 | 78.91 |  |  |  |  |  |  |  |
| **S100** |  |  |  |  |  |  |  |  |  | 99.02 |  |  | 0.18 |  |  |  | 0.27 | 0.54 |  |  |
| **E119** | 0.18 |  |  |  |  |  |  |  | 99.73 |  |  |  |  |  |  |  |  |  | 0.09 |  |
| **K120** |  |  |  |  |  |  | 43.16 | 56.84 |  |  |  |  |  |  |  |  |  |  |  |  |
| **N132** |  | 56.57 |  |  |  |  |  |  |  |  |  | 0.09 |  |  |  |  | 0.09 | 43.07 | 0.09 |  |
| **D166** |  |  |  |  |  |  |  |  |  |  |  |  |  |  |  |  |  |  | 100 |  |
| **N169** | 77.12 | 0.27 |  |  |  |  |  |  |  | 3.66 |  |  |  |  |  |  | 0.09 | 18.59 | 0.27 |  |
| **K191** |  |  |  |  |  |  | 99.91 |  | 0.09 |  |  |  |  |  |  |  |  |  |  |  |
